# Leveraging School Nutrition Environments Through Healthy Universal School Meals Laws to Improve Child Health and Academic Outcomes in the United States

**DOI:** 10.3390/nu18061001

**Published:** 2026-03-21

**Authors:** Olivia M. Thompson, Kathryn E. Coakley

**Affiliations:** Health Sciences Center, College of Population Health, The University of New Mexico, 1155 University Blvd. S, Albuquerque, NM 87131, USA; kecoakley@salud.unm.edu

**Keywords:** healthy universal school meals, food insecurity, hunger, childhood obesity, National School Lunch Program, School Breakfast Program, Community Eligibility Program, education policy, nutrition policy, Healthy, Hunger-Free Kids Act of 2010

## Abstract

**Background/Objectives**: The purpose of this article (a comparative analysis of state laws) is to thoroughly examine enacted state-level healthy universal school meals bills to summarize bill content and determine current practices for program implementation and long-term viability, with special attention to the Community Eligibility Provision (CEP). **Methods**: Bills enacted at the state level, as of 31 December 2025, were located electronically on state legislature websites and subsequently reviewed with rules, regulations, and implementation guidelines. Content analyses were conducted to identify patterns, themes, and key concepts pertaining to healthy universal school meals laws and program implementation guidelines to inform comparison policy analyses. **Results**: Nine states (California, Colorado, Maine, Massachusetts, Michigan, Minnesota, New Mexico, New York, and Vermont) have healthy universal school meals laws that include mandatory funding provisions for programming. Michigan is the only state that has a non-permanent law. Such laws eliminate requirements to certify individual students for free, reduced-price, or full-price meals based on their household income, and instead allow entire schools and/or school districts to offer all enrolled students no-cost meals. All states are funding healthy universal school meals programming by leveraging existing or new tax revenue to bridge the gap between the cost of school meals and federal meal reimbursements. **Conclusions**: State laws that leverage the Community Eligibility Provision (CEP) have become a key way to sustain universal school meal programs when federal funding falls short. States that direct resources to high-poverty schools, help districts determine the most accurate Identified Student Percentage, and reduce undercounting through strong direct-certification practices are better positioned to maintain universal meals over time. These strategies strengthen both child health and academic outcomes by ensuring stable access to no-cost, nutritious meals.

## 1. Introduction

School nutrition environments are among the most important determinants of child health. Not only do most children spend between 170 and 180 days in school annually [1], but on these days, they may consume breakfast, lunch, snacks, and/or dinner while at school [2] at low or no cost because of state and/or federal school meal subsidies and/or meal reimbursements [2,3,4,5,6,7,8,9,10,11,12,13,14,15,16,17,18,19,20]. For example, in approximately 94,000 United States schools, School Food Authorities operate the National School Lunch Program (NSLP) and/or the School Breakfast Program (SBP) [2]. Students who participate in these programs are provided with lunch and/or breakfast meals at no cost, or they can purchase meals at a reduced cost or pay full price. Students are eligible to receive a reduced-price or free school meal if either (1) their household income is between 130% and 185% of the federal poverty level (reduced-price meal eligible) or at or below 130% of the federal poverty level (free meal eligible) or (2) their school or school district qualifies for participation in the Community Eligibility Provision (CEP), or in another like federal provision, such as Provision 2 [21,22]. While NSLP and SBP meal prices vary by state, school district, and school levels; across the country, students who pay a full price typically spend between $3.00 and $3.90 for lunch and $1.90 and $2.00 for breakfast, and students who pay a reduced price typically spend $0.40 for lunch and $0.30 for breakfast [23]. School Food Authorities that participate in the NSLP and/or SBP are provided with federal reimbursements per meal for lunches and/or breakfasts served. Federal reimbursement rates for lunch meals for the 48 contiguous states range between $0.42 and $0.59 for a full-price lunch, $4.03 and $4.29 for a reduced-price lunch, and $4.43 and $4.69 for a free lunch [2]. Federal reimbursement rates for breakfast meals for the 48 contiguous states are $0.39 for a full-price breakfast, and range between $2.07 and $2.54 for a reduced-price breakfast and $2.37 and $2.84 for a free breakfast [2].

The CEP, a federal non-pricing meal service option for School Food Authorities authorized under the Healthy, Hunger-Free Kids Act of 2010 [24], allows high-poverty schools and school districts that participate in both the NSLP and SBP to provide lunch and breakfast meals at no cost to all enrolled students, regardless of their ability to pay [22]. A high-poverty school or school district is one with an Identified Student Percentage (ISP) of at least 25%; in other words, it is one whereby at least 25% of enrolled students are either categorically or directly eligible for no-cost meals without a household application for free or reduced-price meals [21,22]. A student is categorically eligible for no-cost meals if the student is homeless, in foster care, a runaway, a migrant, or enrolled in a Head Start program [22]. A student is directly eligible for no-cost meals if the student or a household member participates in Medicaid (for Medicaid Demonstration Project states only) or is enrolled in a federal food assistance program such as the Supplemental Nutrition Assistance Program (SNAP), the Temporary Assistance Program for Needy Families (TANF), or the Food Distribution Program on Indian Reservations (FDPIR) [22]. Unlike schools and/or school districts that participate in the NSLP and/or SBP but do not participate in the CEP, CEP schools and/or school districts are federally reimbursed for meals served at either free or PAID rates (not reduced-price and full-price rates), depending on the percentage of enrolled students who are either categorically or directly eligible for no-cost meals [22]. Free-meal reimbursement rates are provided for students who are either categorically or directly eligible for no-cost meals, and PAID meal reimbursement rates are provided for students who are neither categorically nor directly eligible for no-cost meals [22]. The meal reimbursement formulas incorporate a federally determined multiplier of 1.6 and are as follows: Free Meal Percentage = ISP X 1.6 and PAID Meal Percentage = 100% − (ISP X 1.6) [22].

A CEP school and/or school district must have an ISP of 62.5% or higher to have 100% of its meals reimbursed at the free rate. If a CEP school and/or school district has an ISP between 25% and 62.5%, its meals will be reimbursed using a combination of free and PAID rates but, importantly, not reduced-price rates. For example, a school with an ISP of 25% would have 40% of its meals reimbursed at the free rate (25% × 1.6 = 40%) and 60% of its meals reimbursed at the PAID rate (100% − [25% × 1.6]), while a school with an ISP of 62.5% would have 100% of its meals reimbursed at the free rate (62.5% × 1.6 = 100%). The PAID reimbursement rate for the 48 contiguous states is $0.40 for breakfast and $0.44 for lunch [25], which is much lower than the reduced-price rate for both types of meals (i.e., the reduced-price federal reimbursement rate typically exceeds $2.00 for breakfast and $4.00 for lunch). Thus, for schools and/or school districts that have ISPs between 25% and 62.5% (chiefly for those with ISPs at or near 25%), it may be financially advantageous for School Food Authorities to qualify students individually for school meals (as opposed to participating in the CEP) so that they may pull down free, reduced-price, and full-price meal reimbursement rates and not just free and PAID reimbursement rates. This financial reality could dampen a School Food Authority’s ability to offer no-cost meals to all enrolled students unless state and/or local funds are available to cover any funding gaps.

States and individual schools and/or school districts may elect to provide funding for no-cost meals for all enrolled students by dedicating monies to support school meal programs; however, this strategy is rarely utilized by individual schools and school districts but instead utilized by states. As of 31 December 2025, nine states (i.e., California, Colorado, Maine, Massachusetts, Michigan, Minnesota, New Mexico, New York, and Vermont) have healthy universal school meals laws that allocated (and continue to allocate) funds for no-cost school breakfast and lunch meals for all children, regardless of their ability to pay [3,4,5,6,7,8,9,10,11,12,13,14,15,16,17,18,19,20]. Mounting evidence suggests that healthy universal school meals laws have positive impacts on both school- and student-level outcomes in particular [26,27]. In a review by Cohen et al. [26], healthy universal school meals program implementation was demonstrated to improve diet quality, food security, and academic performance measures among youth; and in a review by Spill et al. [27], healthy universal school meals program implementation was demonstrated to increase student participation in both school lunch and breakfast programs, as well as school attendance, and decrease childhood obesity prevalence, as well as the number of school suspensions [26,27]. Moreover, while the federal funding structure for school meals has not changed in the nine states that have adopted healthy universal school meals laws and implemented programming, eight of the nine states (i.e., California, Colorado, Massachusetts, Michigan, Minnesota, New Mexico, New York, and Vermont) require School Food Authorities to adopt the CEP (or a like federal provision, such as Provision 2) in order to maximize federal funding whenever possible [3,4,5,6,7,8,9,10,11,12,13,14,15,16,17,18,19,20,28]. In some of these eight states’ schools and school districts, however, it could be financially advantageous for School Food Authorities to forgo participating in the CEP and instead qualify students individually for free, reduced-price, or full-price meals, thereby creating a potential financial disadvantage for well-intended states or for themselves, and consequently having to scale back or even cancel beneficial programming. Importantly, very little comparative work as to which state policy models (funding formulas, CEP mandates, technical assistance, and local foods incentives) are most sustainable and resilient to federal or economic shocks has been conducted. The purpose of this article (a comparative analysis of state laws) is to thoroughly examine enacted state-level healthy universal school meals bills and Supplementary Materials to summarize content and determine current practices for program implementation and long-term viability, with special attention to the CEP.

## 2. Materials and Methods

Healthy universal school meals bills enacted at the state level between 1 January 2021, and 31 December 2025 were located electronically on state legislature websites [3,4,5,6,7,8,9,10,11,12,13,14,15,16,17,18,19,20] and subsequently reviewed by study authors, along with any Supplementary Materials (i.e., rules, regulations, and implementation guidelines). Thereafter, the authors conducted a comparative policy analysis [29] to identify current practices for programming at school and/or school district levels across the United States. State-level demographic data, such as state name, controlling political party affiliation, economic data, race/ethnicity data, education data, health data, and CEP participation data, were collected from individual state’s governmental websites [3,4,5,6,7,8,9,10,11,12,13,14,15,16,17,18,19,20] by the United States Census Bureau through the 2024 American Community Survey [30] and by the Food Research and Action Center [31] and are presented in Table 1. Of note, economic data included percentage of both children and all people in poverty, household median income, and percentage of persons aged 16 and older employed, which is a employment–population ratio; race/ethnicity data were categorized as white, non-Hispanic and any race, and Hispanic; education data included percentage of adults with at least a bachelor’s degree; and health data included percentage of persons without healthcare coverage). Bill-level variables such as year introduced, year implemented, funding required (Yes/No), participation required (Yes/No), CEP participation required (Yes/No), ISP percentage required for CEP participation, grades allowed to participate (k-12, pre-k, and other), grants program(s) offered (i.e., local foods, infrastructure, and/or education and training), and other provisions required or encouraged (Yes/No) were identified, coded, and incorporated into a data table by study authors for ease of use (see Table 2). Other provisions were coded as local foods procurement (defined as items grown, processed, and sold within each state), scratch cooking (defined as preparing meals using raw, unprocessed, or minimally processed ingredients), culturally relevant meals (defined as meals that align with students’ heritage), family involvement and/or advisory committee (defined as having a parent and/or community advisory committee), adequate opportunity/ample time to eat (defined broadly, but generally meaning at least 20 min of seated time for lunch and 10 min of seated time for breakfast), food donation (defined as setting aside intact foods such as fruits or unopened containers for donation to area food banks or charities), enhanced nutrition (defined as making food more nutrient-dense), enhanced technical support (defined as providing technical support for healthy universal school meal programming specifically), food waste reduction (defined as the practice of minimizing the amount of edible food discarded or lost through methods such as composting, offer vs. serve strategies, share tables, etc.), and meal debt forgiveness (defined as cancelling or paying off the outstanding school meal balances owed by families). Bill and Supplementary Material content was analyzed qualitatively through manual analyses to identify patterns, themes, and key concepts pertaining to healthy universal school meals laws and programming for incorporation into a plain-language executive summary organized by state.

## 3. Results

As of 31 December 2025, nine states (i.e., California, Colorado, Maine, Massachusetts, Michigan, Minnesota, New Mexico, New York, and Vermont) have adopted healthy universal school meals laws and have implemented related programming at school and/or school district levels. As shown in Table 1, all of the nine states with enacted universal school meals laws are (senate) Democratic Party-controlled as of 31 December 2025. In all states but New Mexico and California, the predominate race/ethnicity was non-Hispanic white, and the economic and education data demonstrated that (1) the poverty percentage for all people ranged from 9.0% (Vermont) to 16.4% (New Mexico); (2) the poverty percentage for children ranged from 9.1% (Vermont) to 17.9% (New Mexico); (3) the median household income was as low as $67,816 (New Mexico) and as high as $104,828 (Massachusetts); (4) the employment rate was as low as 54.8% (New Mexico) and as high as 65.5% (Minnesota); and (5) the percentage for adults with at least a college education was as low as 31.8% (New Mexico) and as high as 48.3% (Massachusetts). Moreover, health data demonstrated that the percentage of adults without health insurance was as low as 2.8% (Massachusetts) and as high as 10.1% (New Mexico). With regard to CEP participation, data demonstrated that participation was at or near 100% for all states except for Maine and Minnesota (see Table 1).

As described below in state-specific executive summaries and shown in Table 2, two of the nine states with healthy universal school meals laws (i.e., Colorado and Michigan) do not require individual schools and/or school districts to participate in their programs, while the remaining seven states require participation for at least all public schools and/or school districts. Only Maine does not require participation in the CEP or a like federal provision, such as Provision 2; however, California, unlike the other states that do require CEP participation, only requires CEP participation if a school or school district’s ISP is 40% or greater. Only Colorado and Michigan allow students in grades other than pre-k-12 to participate in programming, and only California, Colorado, New Mexico, and Vermont offer funds for local foods, infrastructure, and/or education and training grants programs. Miane and Minnesota were the only states that did not require or encourage some other healthy universal school meals program provision (see the executive summaries and Table 2).

### 3.1. California

California’s Universal Meals Program was established by statute in 2021 for implementation beginning in the 2022–2023 school year through Assembly Bill 130, and later, in 2023, it was strengthened through enactment of Senate Bill 348 [3,4]. California’s program, one that is required for all public schools and/or school districts, charter schools, and county offices of education that participate in the NSLP and SBP, provides meal reimbursements, as well as education, training, and infrastructure funds [3]. All participating entities are required to provide free breakfast and lunch meals to all students enrolled in kindergarten through 12th grade (qualified pre-k students can also participate) and are encouraged to apply for grants for education, training, and/or infrastructure items such as kitchen equipment [3]. Furthermore, while all participating schools and/or school districts are reimbursed by the state for all qualifying meals at the federal free-priced rate, high-poverty schools and/or school districts must participate in the CEP (or a like federal provision, such as Provision 2) before utilizing state funds if their ISP is 40 percent or greater [5]. Universal Meals Program funding is subject to annual budget appropriations from California’s Proposition 98 (Education) General Fund [3,4,5]. Of note, California’s program requires that (1) students are provided with adequate time to eat; (2) foods with a higher nutritional density are prioritized when there is added sugar or sodium in the food; and (3) if the NSLP or SBP allows more added sugar or sodium than is recommended by the most recent Dietary Guidelines for Americans, state agencies and partners develop maximum daily added sugar intake recommendations in line with the American Academy of Pediatrics’ standards for children two years of age and older and maximum daily added sodium intake recommendations in line with recommendations for children and adolescents in the Dietary Guidelines for Americans [4].

### 3.2. Colorado

Colorado’s Healthy School Meals for All program was established by statute in 2022 through House Bill 22-1414, which referred Proposition FF to the ballot for voter approval. Later, House Bill 25-1274 was passed in 2025, which likewise referred Proposition LL to the ballot to raise additional program revenue by lowering tax deduction limits defined in Proposition FF [6,7]. Colorado’s program, a voluntary one that began in the 2023–2024 school year, provides meal reimbursements, local foods purchasing grants, and stipends for school foodservice staff to any public school and/or school district, charter school, day treatment facility, or Residential Child Care Institution that participates in the NSLP and SBP and chooses to opt in [6]. All participating schools and/or school districts are required to provide free breakfast and lunch meals to all students enrolled in kindergarten through 12th grade (qualified pre-k students can also participate) and are encouraged to (1) establish advisory committees to advise on gathering student feedback on meals and meal preferences; (2) consider culture in recipe development; (3) conduct taste-tests; (4) reduce food waste; (5) increase scratch cooking; and apply for local food purchasing grants and stipends for school food-service staff [6]. Furthermore, all participating schools and/or school districts are reimbursed by the state for all qualifying meals at the federal free-priced rate. High-poverty schools and/or school districts, in particular, are required to participate in the CEP (or a like federal provision, such as Provision 2) before utilizing state funds [6]. Healthy School Meals for All program funding is subject to annual budget appropriations from Colorado’s Healthy School Meals for All Cash Fund (see House Bill 24-1390) and, if necessary, Education Fund [6,7].

### 3.3. Maine

Maine’s healthy universal school meals program was established by statute in 2022 for implementation beginning in the 2022–2023 school year through Senate Paper 540/Legislative Document 1679 [8]. Maine’s program, one that is required for all public schools and/or school districts that participate in the NSLP and SBP, provides meal reimbursements [8]. All participating schools and/or school districts are required to provide free breakfast and lunch meals to all students enrolled in kindergarten through 12th grade (pre-k students can participate if they are located within school buildings) [8]. Furthermore, all participating schools and/or school districts are reimbursed by the state for all qualifying meals at the federal free-priced rate. However, there is pending legislation to require all participating schools and/or school districts that are high poverty to maximize federal funding through the CEP [9]. As of the writing of this article, CEP-eligible schools and/or school districts are not required to apply for the CEP to partake in healthy universal school meals programming [8]. Healthy universal school meals program funding is subject to annual budget appropriations from, primarily, Maine’s General Fund [8].

### 3.4. Massachusetts

Massachusetts’s Free Universal School Meals program was established by statute in 2023 for implementation beginning in the 2023–2024 school year through the state’s Fiscal Year 2024 Budget Bill, the relevant part of which originated from House Bill 603 and Senate Bill 261 [10]. Massachusetts’s program, one that is required for all public schools and/or school districts and charter schools that participate in the NSLP and SBP, provides meal reimbursements [10]. All participating schools and/or school districts are required to provide free breakfast and lunch meals to all students enrolled in kindergarten through 12th grade (qualified pre-k students can also participate) [10]. Furthermore, while all participating schools and/or school districts are reimbursed by the state for all qualifying meals at the federal free-priced rate, high-poverty schools and/or school districts must participate in the CEP (or a like federal provision, such as Provision 2) before utilizing state funds [10]. Free Universal School Meals program funding is subject to annual budget appropriations from both Massachusetts’s Education and Transportation Funds, which are sustained by revenue from the Fair Share Amendment or 4% tax on annual income exceeding $1 million [10]. Of note, Massachusetts’s program requires that all students be provided with a breakfast meal after the beginning of the instructional day using a service model that best suits its students (e.g., breakfast in the classroom, grab-and-go breakfast, and/or second chance breakfast) [10].

### 3.5. Michigan

Michigan’s healthy universal school meals program, known as the Michigan School Meals Program, was established by (non-permanent) statute in 2024 for implementation beginning in the 2023–2024 school year through the state’s Fiscal Year 2024 Budget Bill (Enrolled House Bill 5507) [11]. Michigan’s program, a voluntary one that is dependent on annual budget renewals, provides meal reimbursements to any public school and/or school district and charter school that participates in the NSLP and SBP that chooses to opt in [11]. All participating schools and/or school districts are required to provide free breakfast and lunch meals to all students enrolled in qualified state-funded preschool programs, adult (up to age 26) special education programs, and kindergarten through 12th grade [11]. Furthermore, while all participating schools and/or school districts are reimbursed by the state for all qualifying meals at the federal free-priced rate, high-poverty schools and/or school districts are required to participate in the CEP (or a like federal provision, such as Provision 2) before utilizing state funds [11]. Michigan School Meals Program funding is subject to annual budget appropriations and renewal from Michigan’s School Aid Fund [11] (see Table 2). Of note, Michigan requires that all participating entities forgive all school meal debt related to federally reimbursable meals before state fund utilization and encourages all participating entities to offer meals that meet students’ dietary restrictions, including the provision of gluten-free meals; vegetarian meals; vegan meals; and, upon request, kosher meals, halal meals, and meals meeting any confirmed allergy restrictions [11].

### 3.6. Minnesota

Minnesota’s Free School Meals program was established by statute in 2023 for implementation beginning in the 2023–2024 school year through House File 5. Later, in 2025, House File 2201 was proposed (the current status is pending) to cap participation for lunch meals such that only households with annual incomes at or below 500% of the federal poverty guidelines (e.g., $156,000 for a family of four) could qualify [13,14,15]. Minnesota’s program, one that is required for all public schools and/or school districts that participate in the NSLP and SBP, provides meal reimbursements [13]. All schools and/or school districts are required to provide free breakfast and lunch meals to all students enrolled in kindergarten through 12th grade (qualified pre-k students can also participate) [13]. Furthermore, while all participating schools and/or school districts are reimbursed by the state for all qualifying meals at the federal free-priced rate, high-poverty schools and/or school districts must participate in the CEP (or a like federal provision, such as Provision 2) before utilizing state funds [13]. Free School Meals program funding is subject to annual budget appropriations from Minnesota’s General Fund [13].

### 3.7. New Mexico

New Mexico’s Healthy Universal School Meals program was established by statute in 2023 for implementation beginning in the 2023–2024 school year through Senate Bill 4 [16]; and later, in 2025, it was strengthened by adopting a rule that establishes the standards and procedures for its implementation [17]. New Mexico’s program, one that is required for all public schools and/or school districts that participate in the NSLP and SBP and voluntary for charter schools, Board of Indian Education schools, tribally controlled schools, and private schools that also participate in the NSLP and SBP, provides meal reimbursements and funding for infrastructure supports and local food procurement [16,17]. Meal reimbursements are performance-based, such that schools and/or school districts that meet meal quality and food waste program implementation standards receive the greater of one thousand dollars or an amount equal to 10 cents multiplied by the number of lunches qualified for federal free meal reimbursement served to students during the preceding school year [17]. All schools and/or school districts are required to provide free breakfast and lunch meals to all students enrolled in kindergarten through 12th grade (qualified pre-k students can also participate) [16]. Furthermore, while all participating schools and/or school districts are reimbursed by the state for all qualifying meals at the federal free-priced rate, high-poverty schools and/or school districts must participate in the CEP (or a like federal provision such as Provision 2) before utilizing state funds [16]. The local foods grant program, while small, provides seed money for the purchase of New Mexican-grown, raised, or processed foods and food products, with a minimum of 75% of funds used to purchase unprocessed and minimally processed products, and with up to 25% of funds used to purchase value-added processed products [17]. Healthy Universal School Meals program funding is subject to annual budget appropriations from New Mexico’s General Fund [16,17]. Of note, meal quality implementation standards require (1) purchasing New Mexico-produced foods or food products; (2) freshly preparing meals using scratch- or semi-scratch-cooking methods; (3) providing culturally relevant meals; and (4) engaging students and their families in choices for menu development. Food waste implementation standards are (1) students in grades k-5 shall be allowed to have up to twenty minutes of seated lunch, and (2) share tables shall be provided, whereby allowable food shall be donated to students, food banks, or other nonprofit charitable organizations each school day [17].

### 3.8. New York

New York’s Universal Free Meals program was established by statute (through amendment of the state’s Education Law Section 915-a) in 2025 for implementation beginning in the 2025–2026 school year through its Fiscal Year 2026 Executive Budget [18]. New York’s program, one that is required for all public schools and/or school districts, charter schools, and non-private schools that participate in the NSLP and SBP, provides meal reimbursements [18]. All schools and/or school districts are required to provide free breakfast and lunch meals to all students enrolled in kindergarten through 12th grade (pre-k students can participate if they are located within school buildings or enrolled in a qualified community-based organization) [18]. Furthermore, while all participating schools and/or school districts are reimbursed by the state for all qualifying meals at a rate for each meal served that equals the combined state and federal reimbursement rate for a free meal for the current school year, high-poverty schools and/or school districts must participate in the CEP (or a like federal provision, such as Provision 2) before utilizing state funds [18]. New York provides schools and/or school districts with technical assistance to promote SNAP enrollment and also to assist in the transition to universal school meals to ensure successful program operations and to maximize federal funding [18]. Universal Free Meals program funding is subject to annual budget appropriations from New York’s General Fund [18].

### 3.9. Vermont

Vermont’s Universal School Meals program was introduced first as a one-year pilot in the 2022–2023 school year through Senate Bill 100 and then enacted permanently in 2023 through House Bill 165 [19,20]. Implemented in the 2023–2024 school year, Vermont’s program, one that is required for all public schools and/or school districts and optional for qualified private schools that participate in the NSLP and SBP, provides meal reimbursements and local foods purchasing grants [20]. All participating schools and/or school districts are required to provide free breakfast and lunch meals to all students enrolled in kindergarten through 12th grade (qualified pre-k students can also participate), and are encouraged to apply for local foods grants [20]. Furthermore, while all participating schools and/or school districts are reimbursed by the state for all qualifying meals at the federal free-priced rate, high-poverty schools and/or school districts are required to participate in the CEP (or a like federal provision such as Provision 2) before utilizing state funds, which are provided through a universal meals supplement [20]. Local foods grant program funds between $0.15 and $0.25 per reimbursable school lunch served are provided to recipient schools and/or school districts [20]. Universal School Meals program funding is subject to annual budget appropriations from Vermont’s Education General Fund and, if necessary, General Fund [20]. Of note, Vermont’s program requires that time spent by students consuming school meals during class is counted as instructional time and emphasizes the implementation of strategies shown to increase student participation in school meal programs (i.e., providing grab-and-go breakfast meals, making breakfast available to students in classrooms after the start of the school day, and collaborating with the schools’ wellness community advisory councils in planning school meals) [20].

**Figure 1:**
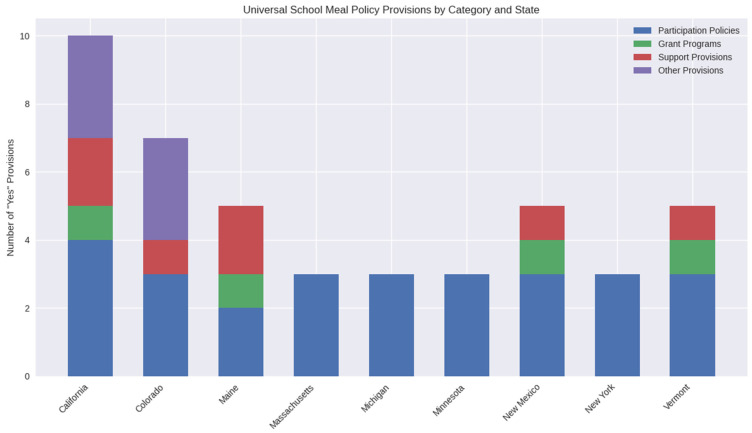
Distribution of “Yes” responses across four healthy, universal school meals categories—Participation Policies, Grant Programs, Support Provisions, and Other Provisions—by state.

As demonstrated in Figure 1, the stacked graph reflects the following four-category structure: (1) participation policies form the base layer because they are the most consistently adopted across states, especially universal pre-K participation and CEP requirements; (2) grant programs add a small but meaningful layer, with only a few states offering local foods procurement and/or training grants, for example; (3) support provisions create visible variation, highlighting differences in operational commitments, such as scratch cooking, adequate time to eat, and/or family engagement; and (4) other provisions—enhanced nutrition, technical support, and food waste reduction—cluster in a few states, making their contribution stand out at the top of the stack. The visualization shows how each state’s healthy universal school meals laws are distributed across participation requirements, grant supports, operational supports, and broader system-level provisions. California and Colorado show tall, multi-layered stacks, indicating broad policy coverage across all four categories. Maine, New Mexico, and Vermont show a distinctive support-provision profile, with grant programs, and policies and procedures designed to increase program participation. Massachusetts, Michigan, Minnesota, and New York cluster with moderate participation layers and minimal additional provisions (see Figure 1).

## 4. Discussion

As of 31 December 2025, nine states have enacted and at least 28 states have considered enacting healthy universal school meals bills [32]. All states, with the exception of Michigan, have structurally similar healthy universal school meals laws that require annual funding for meal reimbursements especially, subject to the availability of funds; however, across each state, there exists nuances in the bill language, budget documents, and program implementation guidance documents. Furthermore, seven of the nine states with enacted healthy universal school meals bills require that all eligible schools and/or school districts participate in programming, while two states (Colorado and Michigan) allow schools and/or school districts to opt in. Eight of the nine states (except Michigan) require that all high-poverty schools participate in the CEP or a like federal provision, and one of these eight states (California) requires that high-poverty schools and/or school districts with an ISP of 40% or greater participate in the CEP or a like federal provision. One state (Maine) encourages but does not require participation in the CEP or a like federal provision. Five states (California, Colorado, Massachusetts, New Mexico, and Vermont) require or encourage providing ample opportunity and adequate time for students to eat meals and/or other important activities, including local food purchasing, scratch or semi-scratch cooking, and engaging students and families in meal and menu development, among others, while the remaining four states are primarily focused on meal reimbursements or drawing down federal funds to cover meal costs specifically.

California stands out because, again, it requires that high-poverty schools and/or school districts participate in the CEP only if their ISP is 40 percent or greater, while all other states require CEP participation in their ISP is 25 percent or greater. Furthermore, the state prioritizes that students are provided with adequate time to eat and that meals served are nutrient-dense and minimize added sugars and sodium in particular. Colorado, a state that does not require schools and/or school districts to participate in programming but instead allows them to opt in, prioritizes providing students with local foods and even provides stipends for school service staff to incentivize scratch cooking. Maine, one of the first states to adopt healthy universal school meals laws, focuses on meal reimbursements, and while the state may require CEP participation in the near future, as of 31 December 2025, it does not require schools and/or school districts to maximize federal funds through use of the CEP. Massachusetts’s program is focused on meal reimbursements, but also requires that key provisions be adopted ensuring that breakfast meals are available to students before and after the beginning of the school day. Michigan’s program, while temporary, has established broad criteria for entities choosing to opt in and, interestingly, requires that all participating entities forgive school meal debt in order to receive state funds. Minnesota’s program provides a cautionary tale about funding universal school meals programming, as state budget shortfalls associated with the high cost of meal reimbursements have caused state lawmakers to rethink program eligibility criteria such that only households with annual incomes at or below 500% of the federal poverty guidelines could qualify. New Mexico, one of the poorest states in the nation, has made a significant investment in universal school meals programming by providing meal reimbursements that incentivize schools and/or schools districts that apply high meal quality and food waste standards, along with providing additional monies for infrastructure supports and local food procurement. New York’s program, the most recent one, stands out in that it requires that technical assistance be provided to participating schools and/or school districts for both SNAP and CEP enrollments. Vermont, well-known for its local foods culture, adopted a law true to form in that their healthy universal school meals law provides significant reimbursement for meals made using local foods and/or food products. Taken together, while all but one of the nine aforementioned states have permanent healthy universal school meals laws that require annual funding for meal reimbursements, most lack funding requirements for important infrastructure supports such as kitchen equipment and cold storage, and comprehensive nutrition education and training for students, parents, and, critically, school food-service staff. Moreover, while a handful of states have provided funding for program evaluation purposes, most states have not allocated such funding and thus have not evaluated their programs in scientifically rigorous ways.

Support from law-makers for healthy universal school meal laws is steadily growing, as evidence continues to mount demonstrating positive relationships between and among healthy universal school meals programming and improved student- and school-level health, academic, and economic outcomes [26,27,33,34,35,36,37,38,39,40]. For example, with regard to student-level outcomes, Ramponi et al. (2025) [33] found that healthy universal free school meals programming was associated with higher participation in both the NSLP and SBP during and after the COVID-19 pandemic, based on a natural experiment using school-level data from 2019 to 2024. Specifically, the study found that states adopting programming saw sustained increases in student participation compared to states without such policies, highlighting the role of universal access in reducing barriers and supporting consistent meal uptake. Jones-Smith et al. (2025) [34] found that healthy universal free school meals programming was associated with modest effects on children’s body mass index (BMI), with no clear evidence of increased obesity risk. Localio et al. (2025) [35] found that healthy universal school meals programming was associated with an 11% reduction over five years in youths with high blood pressure, based on a cohort study of more than 155,000 patients across 1052 schools. The findings suggest that healthy universal free school meals may contribute to meaningfully lower hypertension risk among students, reinforcing the programming’s potential health benefits. Moreover, with regard to school-level outcomes, in a study by Long et al. (2021) [40], healthy universal free school meals programming was associated with lower per-meal costs, largely because schools benefited from economies of scale and reduced administrative burdens. At the same time, schools were able to maintain the nutritional quality of meals, showing that cost savings did not come at the expense of healthier food options.

Furthermore, key stakeholders, such as school officials, parents, and the students themselves, have expressed support for healthy universal school meals laws and subsequent programming. Students note that programming increases school meal accessibility and convenience; aligns well with their preference for healthy, freshly prepared meals; and reduces the stigma associated with school meal program participation [39,40]. Parents note that programming improves their child’s social/emotional development, and indicate that benefits such as convenience, saving money, and better school meal dietary quality motivate them to encourage their child to choose school meals over other meal options including competitive foods [34,35]. School officials note that programming not only reduces the stigma associated with school meal program participation, but that it also reduces meal-related burdens experienced by teachers and other school staff [41,42]. Importantly, however, students, parents, and school food-service staff alike have expressed concerns about the quality (particularly the taste) of healthy universal school meal foods and frequently note food waste as an unintended consequence of the programming [42,43,44,45,46].

Limitations of the current study should be acknowledged. This study’s authors searched for enacted healthy universal school meals bills using state-specific websites in the fall of 2025. Therefore, bills introduced during 2026 legislative sessions and beyond are not reflected in this article. Furthermore, while this study’s authors took care to find state-specific rules, regulations, and program implementation guidelines, it is possible that not all Supplementary Materials were found, as sometimes they are not readily publicly available. Also, due to the recent adoption of healthy universal school meals programming (i.e., within about five years), there exists a dearth of peer-reviewed studies that assess program outcomes and impacts. Funders, including state officials, should therefore prioritize longitudinal data collection activities so that the local-, state-, and national-level outcomes and impacts of healthy universal school meals laws and related programming can be elucidated. Future work should incorporate the voices of key stakeholders throughout the food supply chain system and include validated measures of student-level outcomes, such as overweight or obesity statuses, dietary intakes, food insecurity and hunger, physical and mental health statuses, and academic achievements; household-level outcomes, such as food insecurity and hunger, dietary intakes, and economic measures; and school- and/or school district-level outcomes, such as school meal participation, school attendance, and School Food Authority employment and economic measures, including financial gains or losses associated with participation in the CEP.

## 5. Conclusions

In the absence of comprehensive federal government funding for universal school meals programming, adopting state-level healthy universal school meals laws that maximize federal funding through use of the CEP has recently emerged as a leading strategy to uphold both child and community health in the United States [38,39,47], especially during the current political climate [48,49]. States with comprehensive healthy universal school meals laws that mandate state funding provisions for not only meal reimbursements but also for infrastructure support; nutrition education and training for students, parents, and school food-service staff; and farm-to-school activities, including procuring local foods [50,51,52], are likely to gain public support for continued programming. Moreover, states that (1) allow flexibilities for schools and/or school districts to participate in the CEP when it is financially advantageous for both schools and/or school districts and individual states; (2) prioritize funding based on financial need; and (3) provide technical assistance to high-poverty schools and/or school districts to determine the optimal ISP for CEP participation and to reduce undercounting students who are CEP eligible through direct certification are likely to be able to sustain their healthy universal school meals programs in the long-term and thus markedly improve child health and academic achievement outcomes.

## Figures and Tables

**Table 1:** State-level bill-level characteristics.

Characteristic Variable	California	Colorado	Maine	Massachusetts	Michigan	Minnesota	New Mexico	New York	Vermont
**Political Party Control**									
Governor (Party)	Democratic	Democratic	Democratic	Democratic	Democratic	Democratic	Democratic	Democratic	Republican
Senate Control (Party)	Democratic	Democratic	Democratic	Democratic	Democratic	Democratic	Democratic	Democratic	Democratic
House Control (Party)	Democratic	Democratic	Democratic	Democratic	Republican	Split	Democratic	Democratic	Democratic
**Economic Data**									
Poverty (All People) (%)	11.8%	9.6%	10.6%	9.7%	13.4%	9.3%	16.4%	14.0%	9.0%
Poverty (Children) (%)	14.6%	11.5%	12.7%	11.1%	18.0%	9.5%	21.8%	17.9%	9.1%
Median Household Income (USD)	$100,149	$97,113	$76,442	$104,828	$72,389	$87,117	$67,816	$85,820	$82,730
Employment Rate (%)	60.0%	64.9%	60.0%	64.4%	58.8%	65.5%	54.8%	59.5%	63.0%
**Race and Ethnicity Data**									
White, non-Hispanic (%)	33.6%	65.2%	91.3%	67.7%	73.0%	76.0%	36.1%	53.2%	91.0%
Any Race, Hispanic (%)	40.8%	23.2%	2.4%	14%	6.2%	6.7%	49.1%	20.2%	2.7%
**Education Data**									
Adults with ≥ Bachelor’s Degree (%)	38.1%	47.8%	37.1%	48.3%	33.3%	40.0%	31.8%	41.2%	45.1%
**Health Data**									
Without Healthcare Coverage (%)	5.9%	7.9%	5.5%	2.8%	5.1%	5.1%	10.1%	5.0%	4.2%
**CEP Participation Data**									
CEP-Eligible Schools (*n*)	7488	1128	230	1242	2847	1206	793	4015	264
CEP Participation (%)	97%	100%	50%	100%	95%	41%	100%	100%	100%

Economic, Race and Ethnicity, Education, and Health Data were collected through the 2024 American Community Survey by the United States Census Bureau [30]; Community Eligibility Provision (CEP) Participation Data were collected using the CEP participation calculator available through the Food Research and Action Center [31]; and Political Party Control Data were collected from each state’s governmental website(s) [3,4,5,6,7,8,9,10,11,12,13,14,15,16,17,18,19,20].

**Table 2:** Healthy Universal School Meals Bill-Level Characteristics and Program Implementation Guidelines.

Characteristic Variables	California	Colorado	Maine	Massachusetts	Michigan	Minnesota	New Mexico	New York	Vermont
**Bill Information**									
Year Introduced	2021	2022	2022	2023	2024	2023	2023	2025	2023
Years Implemented	2022/2023	2023/2024	2022/2023	2023/2024	2023/2024	2023/2024	2023/2024	2025/2026	2023/2024
**Fund Appropriations**									
Funding Required (Yes/No)	Yes	Yes	Yes	Yes	Yes	Yes	Yes	Yes	Yes
**Enforcement Language**									
Participation Required (Yes/No)	Yes	No	Yes	Yes	No	Yes	Yes	Yes	Yes
**CEP Participation**									
CEP Required (Yes/No)	Yes	Yes	No	Yes	Yes	Yes	Yes	Yes	Yes
**Identified Student Percentage**									
ISP Percent Required (%)	40%	25%	--	25%	25%	25%	25%	25%	25%
**Grades Allowed to Participate**									
K–12 (Yes/No)	Yes	Yes	Yes	Yes	Yes	Yes	Yes	Yes	Yes
Pre-K (Yes/No)	Yes	Yes	Yes	Yes	Yes	Yes	Yes	Yes	Yes
Other (Yes/No)	No	Yes	No	No	Yes	No	No	No	No
**Grant Programs Offered**									
Local Foods Procurement (Yes/No)	No	Yes	No	No	No	No	Yes	No	Yes
Infrastructure (Yes/No)	Yes	No	No	No	No	No	Yes	No	No
Education and Training (Yes/No)	Yes	Yes	No	No	No	No	No	No	No
**Other Provisions**									
Local Foods Procurement (Yes/No)	No	Yes	No	No	No	No	Yes	No	Yes
Scratch Cooking (Yes/No)	No	Yes	No	No	No	No	Yes	No	No
Culturally-Relevant Meals (Yes/No)	No	Yes	No	No	No	No	Yes	No	No
Family Engagement or Advisory Committee (Yes/No)	No	Yes	No	No	No	No	Yes	No	Yes
Ample Opportunity or Adequate Time to Eat (Yes/No)	Yes	No	No	Yes	No	No	Yes	No	Yes
Food Donation (Yes/No)	No	No	No	No	No	No	Yes	No	No
Enhanced Nutrition (Yes/No)	Yes	Yes	No	No	Yes	No	No	No	No
Enhanced Technical Support (Yes/No)	No	No	No	No	No	No	No	Yes	No
Food Waste Reduction (Yes/No)	No	Yes	No	No	No	No	Yes	No	No
Meal Debt Forgiveness (Yes/No)	No	No	No	No	Yes	No	No	No	No

Data were collected through bill language and implementation guideline documents from each state’s governmental website(s) [3,4,5,6,7,8,9,10,11,12,13,14,15,16,17,18,19,20].

## Data Availability

No new data were created.

## Abbreviations

The following abbreviations are used in this manuscript.

CEP	Community Eligibility Provision
ISP	Identified Student Percentage
NSLP	National School Lunch Program
SBP	School Breakfast Program
SNAP	Supplemental Nutrition Assistance Program
TANF	Temporary Assistance Program for Needy Families
FDPIR	Food Distribution Program on Indian Reservations
